# Mindfulness-based intervention helps preclinical medical students to contain stress, maintain mindfulness and improve academic success

**DOI:** 10.1186/s12909-021-02578-y

**Published:** 2021-03-05

**Authors:** Luisa Charlotte Lampe, Brigitte Müller-Hilke

**Affiliations:** Core Facility for Cell Sorting & Cell Analysis, Laboratory for Clinical Immunology, University Medical Center Rostock, Rostock, Germany

**Keywords:** Mindfulness based stress reduction, Perceived stress, Scholarly success, Medical students

## Abstract

**Background:**

Stress among students is on the rise during early medical school and has been implicated in poor academic performance. Several methods are being discussed to efficiently reduce stress, among them mindfulness-based interventions. We therefore set out to assess how stress, mindfulness, and academic performance are connected and if an intervention on mindfulness based stress reduction could alleviate stress among medical students and improve their academic achievements.

**Methods:**

A non-randomized controlled trial including 143 medical students in their preclinical years was performed in 2019. The students completed two surveys - one in the first, the other in the third term - recording perceived stress and mindfulness via validated scales (PSS-10 and MAAS). In between both, 41 students participated in a voluntary mindfulness-based intervention including six two-hours courses. 86 students served as controls. Scholarly success was assessed via the scores achieved in six exams written during the observation period.

**Results:**

Stress was inversely related with mindfulness and with the results of the most challenging exam. The intervention on mindfulness based stress reduction helped to contain stress and maintain mindfulness during the observation period and this effect lasted for at least six months beyond completion of the intervention. In contrast, beneficial effects on scholarly success were transient and only detectable at completion of the intervention.

**Conclusion:**

Our observation of short- and intermediate term effects resulting from six individual interventions on mindfulness based stress reduction is encouraging and calls for alternative strategies to induce long-lasting impacts.

**Supplementary Information:**

The online version contains supplementary material available at 10.1186/s12909-021-02578-y.

## Background

The WHO defines health not only as a physical but also as a mental and social well-being [1]. Among the conditions that have a considerable impact on mental health, stress is an important one and has been related to common disorders like depression, work-related fatigue burnout and anxiety disorders [2]. These mental disorders are also common in physicians and were shown to affect performance and patients outcomes and to be related to unprofessional behavior [3]. However, already students at an early stage of medical school experience stress, depression and anxiety and an increase thereof in the years to come [4, 5]. Constant academic pressure, increased workloads, financial problems, sleep deprivation, exposure to patients’ suffering and death in combination with a “hidden curriculum” of cynicism may then result in a vicious circle of stress and even more stress which in the long run leads to a decline of mental health [4, 6].

High amounts of stress not only foster the abuse of substances like alcohol and nicotine but can also lead to other unhealthy habits [7, 8]. Moreover, psychological distress among students has been suggested to adversely affect their academic performance [9]. Indeed, stress levels before the first state exam which is taken at the end of the preclinical education were shown to predict academic performance [10]. It is therefore of outmost importance, that instructing young students to become knowledgeable, empathic and professional physicians includes the training of methods for sustained stress reduction.

In this context, mindfulness-based stress reduction has come to the fore. Kabat-Zinn was one of the first to establish mindfulness in Western medicine and described mindfulness as being fully aware of the present moment and of bodily sensations, without being judgmental [11]. Indeed, mindfulness was shown to be inversely associated with depression and stress [12, 13]. And interventions to improve mindfulness led to a decrease in mental distress, anxiety, depression, emotional exhaustion and fatigue inertia while at the same time fostering subjective well-being, empathy and self-compassion [14–17]. These results were observed for early medical students, medical students during their clinical clerkships and primary care physicians alike however, they were assessed directly after completion of the intervention. Longer lasting effects that were still measurable up to 6 or 20 months after the intervention were restricted to a reduction of perceived stress and an increase in self-compassion [18–20]. As of yet, the impact of mindfulness-based stress reduction on academic achievements is less clear. While greater mindfulness was shown to correlate with better academic performance in middle school and bachelor students [21, 22], the information is still lacking whether teaching or improving formal techniques on mindfulness based stress reduction (MBSR) will also improve academic accomplishments of medical students.

The present study therefore aimed to examine the connectedness between stress, mindfulness and academic achievements in early medical students and whether an intervention on mindfulness-based stress reduction would lead to a sustained improvement of both, mental well-being and scholarly success.

## Method

### Design and setting

**Fig. 1:**
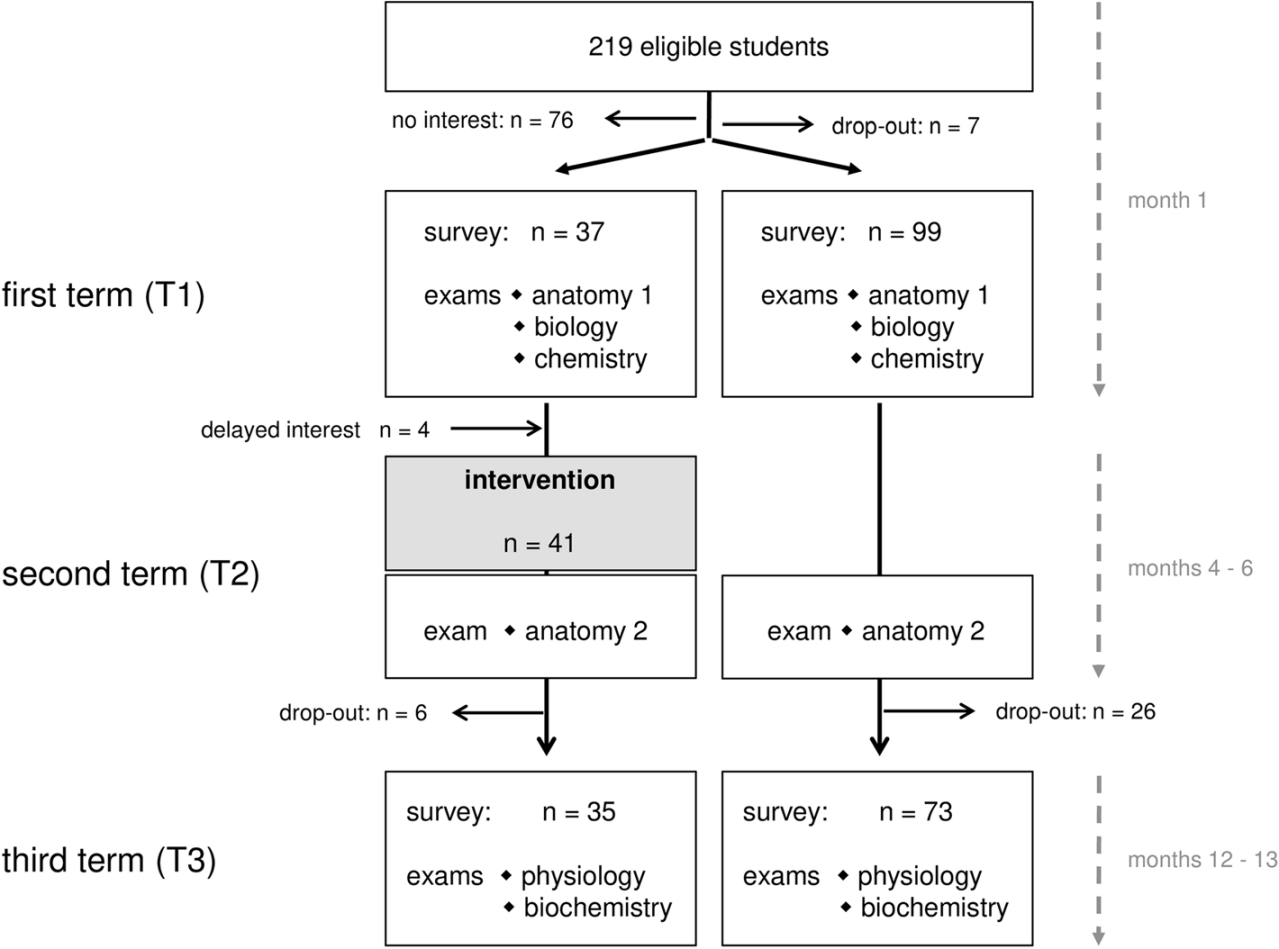
Study design. The flow chart indicates the time points of the study (T1-T3), the actions taken and the numbers of students participating at each stage

We performed a controlled trial on the effect of teaching mindfulness-based stress reduction (MBSR) during the first two preclinical years (term 1 to term 3) of medical school at the Rostock University Medical Center. Recruitment took place in January 2019 and follow-up assessments lasted until January 2020. The study design included two identical online surveys on mindfulness and perceived stress, conducted in January 2019 (T1) and December 2019 (T3), respectively. In between, from April to June 2019 (T2), the students were offered a voluntary intervention on formal techniques of mindfulness. The results of a total of six exams taken around T1 (baseline), T2 (after completion of the intervention) and T3 (6 months later) were collected to assess academic achievements in the course of the study (Fig. 1). These exams were chosen because they included all written exams in the time frame of our study that were summative assessments and covered natural sciences.

### Participant recruitment

During a compulsory course in their first preclinical year, a complete academic year at the Rostock University Medical Center was informed about the study and invited to participate. Participation was voluntary and required a written consent to the monitoring of perceived stress, Mindfulness Attention and Awareness, academic achievements as well as an information on age and gender. At the same time, the students were given notice about a voluntary course during the upcoming term that would cover formal techniques of mindfulness. The study was approved by the ethics committee of the medical faculty of the University of Rostock and is registered under A 2019–0003. Written informed consent was obtained from all participants and all methods were performed in accordance with the relevant guidelines and regulations**.**

### MBSR

The intervention teaching formal techniques of MBSR included 6 two-hours courses. The curriculum was based on the original Mindfulness-Based Stress Reduction developed by Kabat-Zinn, but was adapted to fit our voluntary course. A peer-assisted-learning approach was used whereby the peer was a student two years senior to the class. She had been trained during an 8-weeks MBSR course given by a certified MBSR trainer of ten years. The first two hours started with an introduction into mindfulness, followed by an exercise of mindful breathing. Thereafter, the students were invited to share and reflect their experiences, such as thoughts, feelings and judgments and give a feedback. Also in the first session, groups of 2–3 students each were assigned publications on mindfulness which were to be presented during the remaining sessions. At the end of these first two hours, mindful breathing was once again practiced and additional homework was assigned which included practicing the formal methods of mindfulness at home and being more mindful during everyday activities. The following courses covered sitting meditation, body-scan, eating mindfully and gentle yoga respectively, and had comparable structures: they started with exercises followed by discussion on experiences of the participants with their homework of practicing mindfulness, presentations of the assigned publications and again practicing mindfulness.

### Outcome measures

We assessed perceived stress and mindfulness attention and awareness as mental health outcome measures using validated tools. Academic achievements were assessed by collecting exam results.

### Perceived stress (PSS-10)

Perceived stress was assessed using the German version of the perceived stress scale (PSS-10) provided by Klein [23]. The PSS-10 comprises 10 items which are rated on an endpoint-named LIKERT scale ranging from 0 (`never´) to 4 (`very often´) and investigates how often participants experienced certain events of perceived helplessness or self-efficacy, which are the two dimensions of the PSS.

### Mindfulness (MAAS)

Mindfulness was assessed using the German version of the Mindfulness Attention and Awareness Scale (MAAS) [24]. The MAAS comprises 15 items which are rated on an endpoint-named LIKERT scale ranging from 1 (`almost always´) to 6 (`hardly ever´) and determines how often everyday activities are accomplished without being aware of doing so. Higher results therefore indicate greater mindfulness.

Both questionnaires were compiled online using EvaSys and were sent as one survey to the students by email.

### Academic achievements

The following exam results were collected: anatomy 1, biology and chemistry at T1, anatomy 2 at T2 and physiology and biochemistry at T3.

### Data analysis

**Fig. 2:**
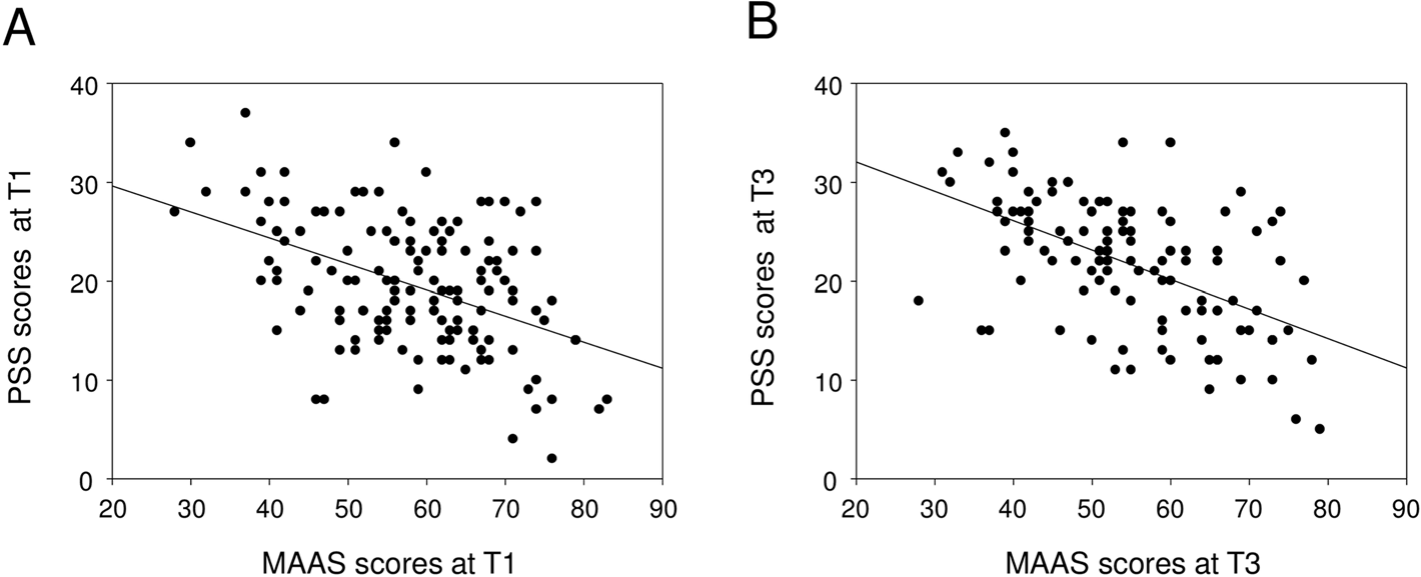
Mindfulness and perceived stress were inversely correlated. Correlations were calculated via Pearson (T1) or Spearman Rank analyses (T3). (A) (r) = − 0.4594, (r) = − 0.4594, the 95% confidence interval = − 0.5831 to − 0.3149, two-tailed *P* value is < 0.0001. (B) r = − 0.5300, the 95% confidence interval = − 0.6552 to − 0.3764, the two tailed P value is < 0.0001. Each dot represents one individual

**Fig. 3:**
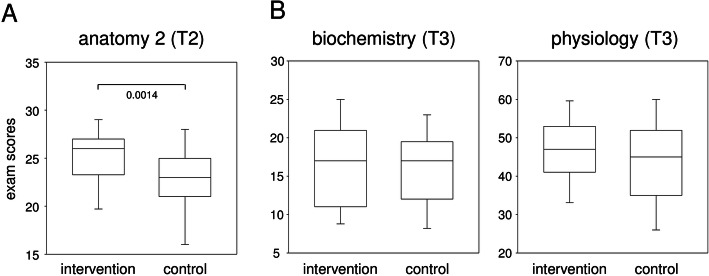
The improvement of academic achievements following an intervention on MBSR was transient. The boxplots show exam scores of intervention and control cohorts monitored at T2 and T3. Mann-Whitney-U tests (A) and t-tests (B) were performed to explore statistically significant differences

**Fig. 4:**
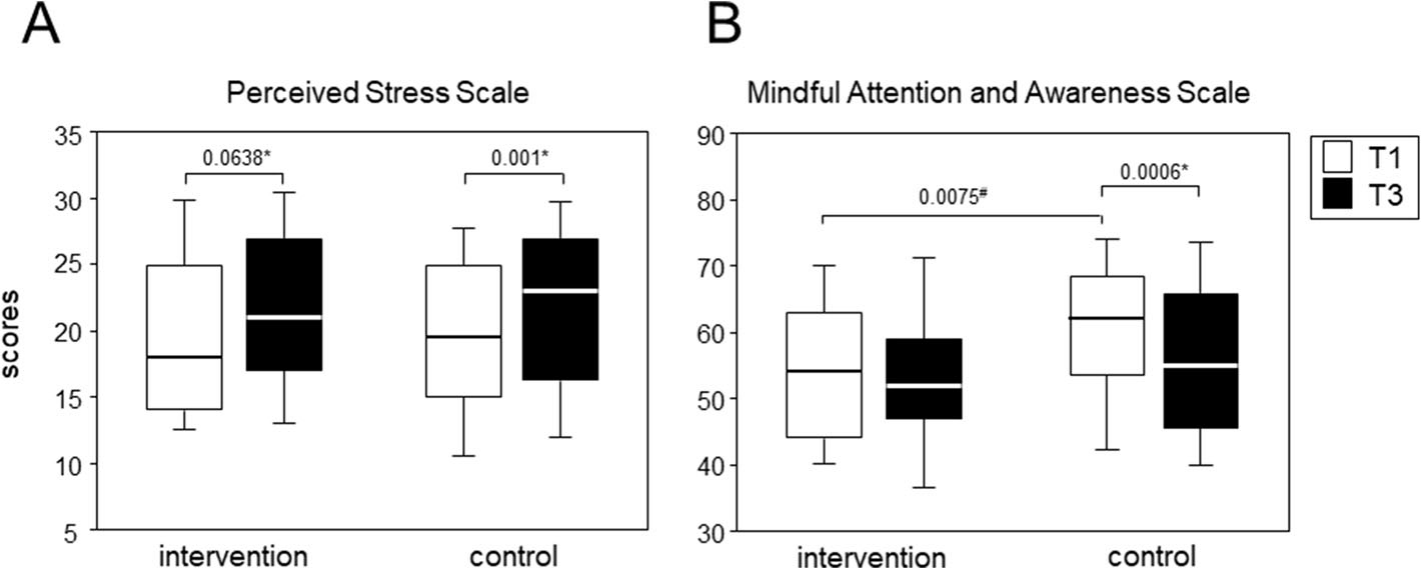
The intervention on MBSR exerted a sustained beneficial effect on mindfulness and perceived stress in medical students**.** The boxplots compare perceived stress (A) and mindfulness (B) in the first (white box) and third (black box) term between intervention and control cohorts The boxes represent medians as well as upper and lower quartiles. The whiskers show 90th and 10th percentiles. *P*-values result from *Wilcoxon matched-pairs signed-ranks and ^#^unpaired t-tests, respectively

All data were tested for Gaussian distribution using the Shapiro-Wilk-test. Correlation analyses were performed via Pearson for data following Gaussian distribution and via Spearman for data not following Gaussian distribution (Fig. 2). Comparisons between intervention and control groups were performed via t-test for data following Gaussian distribution and via Mann-Whitney U test for data not following Gaussian distribution (Fig. 3). For pairwise comparisons, paired t-tests or Wilcoxon matched-pairs signed-ranks-tests were performed, depending on whether data did or did not follow Gaussian distribution, respectively (Fig. 4). For comparisons of female/male ratios, we performed Fisher’s exact tests. Statistical analyses were carried out using IBM SPSS statistics, version 25.

For assessments of reliability and validity, all PSS-10 and MAAS questionnaires returned were included. To assess the reliability, Cronbach’s alphas were calculated. To assess validity, we performed confirmatory factor analyses (CFA) using a two-dimension model for the PSS and a one-dimension model for MAAS in R. The incremental fit was described calculating the comparative fit index (CFI), the Tucker-Lewis Index (TLI) and the Chi-square, respectively. For absolute measure of fit, the root mean square error of approximation (RMSEA) was calculated.

## Results

### Study cohort

Out of the whole year of 219 students, 143 declared their written consent to participate in the study and 136 of these completed the first survey on perceived stress and mindfulness at T1. 41 students volunteered to take the course on mindfulness based stress reduction (MBSR) in the second term (T2). Even though the proportions of females among the students who completed the first survey and the rest of the academic year (those who opted out or had no interest in the study) were significantly different, the MBSR intervention and control cohorts were comparable (Table 1). Of the 136 students who returned the first, 112 students also completed the second survey on perceived stress and mindfulness at T3. Varying numbers of students who sat the upcoming exams resulted from the possibility to postpone exams, even though this implied the risk of delaying academic progress. Figure 1 summarizes the study design and the numbers of students assessed at each time point.

**Table 1:** Demographics of intervention and control cohort

Variable, measure	first survey completed	rest of the academic year	P value
Age [mean / SD]	20.4 / 2.6	20.7 / 3.1	0.9131*
Female gender [n / %]	109 / 80.1	43 / 53.8	< 0.0001^#^
	intervention cohort	control cohort	
Female gender [n / %]	37 / 90.2	72 / 75.8	0.0623^#^

*resulting from Mann-Whitney U-test; ^#^resulting from Fisher’s exact test;

### Confirmation of reliability and validity of the questionnaires

The reliability and validity of the perceived stress (PSS-10) and Mindfulness Attention and Awareness (MAAS) Scales were confirmed by using all data sates that were returned to us (T1: *n* = 139, T3: *n* = 148). Internal consistencies were assessed by calculating Cronbach’s alpha for both scales (PSS and MAAS) and both time points. The resulting values ranged between 0.851 and 0.914 and thus proved reliable. Validities were confirmed via confirmatory factor analyses and the results also showed a good fit (additional Table 1[see additional file 1]). Further descriptive statistics for the questionnaires are provided in the additional Tables 2–5 [see additional file 1].

### Mindfulness and perceived stress were inversely correlated

Our longitudinal analysis of medical students over their first and third term revealed, that elevated mindfulness was paralleled by a reduction of perceived stress and vice versa. Spearman rank correlation analyses performed for our study cohort revealed statistically significant correlations with coefficients of r = − 0.4594 at T1 (95% CI = − 0.5831 to − 0.3149, *p* < 0.0001) and r = − 0.5300 at T3 (95% CI = − 0.6552 to − 0.3764, p < 0.0001) (Fig. 2).

### Neither mindfulness nor perceived stress correlated with academic achievements in the first year at medical school

We here assessed whether mindfulness or perceived stress were associated with exam results in first year medical students and therefore correlated the exam scores achieved at T1 with the scores obtained from the simultaneously assessed stress and mindfulness scales, respectively. While there were no correlations between mindfulness and exam scores, there was a negligible correlation between perceived stress and the results from the biology exam that yielded in a correlation coefficient of 0.177 and a *p*-value of 0.0456.

### The improvement of academic achievements following an intervention on MBSR was transient

In order to analyze whether our intervention teaching formal techniques of MBSR impacted positively on the academic performance, we compared exam results between intervention and control cohorts. Figure 3 shows that indeed, shortly after completion of the intervention at T2, those who participated obtained significantly better results in the anatomy 2 exam than the control cohort as indicated by a p-value of 0.0014. However, these differences were of short term and no longer detectable at T3 when the biochemistry and physiology exams were written.

### The intervention on MBSR extended a sustained beneficial effect in mindfulness and perceived stress in medical students

During their first two years at medical school, students showed a rise in perceived stress as well as a loss of mindfulness. However, while the increase in perceived stress was significant for our control cohort (T1: median = 19, IQR = 10.5, T3: median = 23, IQR = 11.5), the difference between T1 and T3 did not reach statistical significance in the intervention cohort (T1: median = 18, IQR = 11, T3: median = 21, IQR = 10) (Fig. 4a). Likewise, mindfulness showed no significant decrease in the intervention cohort (T1: median = 54, IQR = 19, T3: median = 52, IQR = 12), whereas the control cohort experienced a significant drop (T1: median = 62, IQR = 15, T3: median = 55, IQR = 20.5). Of note, the beneficial effect of our intervention persisted for at least six months as the intervention on MBSR was completed in June while perceived stress and mindfulness were assessed in the following December (Fig. 4b). Interestingly, at T1 the intervention cohort showed significantly less mindfulness than the control cohort.

### Increased stress in the course of the academic progress impacted negatively on exam results

Table 2 shows that the various exams have different degrees of difficulty with biochemistry and physiology at T3 including the most difficult questions as assessed via item difficulty. However, while 40% of correct answers sufficed to pass biochemistry, all the other exams including physiology required 60%. Among students, the physiology exam is therefore considered the most challenging one. While at T3 there were still no correlations between mindfulness or the loss thereof on the one hand and academic achievements on the other. Likewise, there was no correlation between perceived stress and the exam results in biochemistry. However, stress was inversely related to exam results in physiology, resulting in a correlation coefficient of r = − 0.3364, a 95% CI of − 0.4997 to − 0.1501 and a corresponding *p*-value of 0.0004.

**Table 2:** Comparison of exam metrics yield physiology as the most challenging exam

	max. Score	average item difficulty*	passed (%)
anatomy (T1)	30	0.716	82.6
biology (T1)	40	0.684	74.4
chemistry (T1)	63	0.672	87.9
anatomy (T2)	30	0.741	85.4
biochemistry (T3)	34	0.459	69.7
physiology (T3)	80	0.522	32.8

*assessed as average score/maximum score

## Discussion

We here analyzed the connectedness between stress, mindfulness and academic achievements in early medical students and obtained several results, some of which were to be expected. Firstly, mindfulness and perceived stress showed a significant negative correlation. This is in line with previous studies [9, 12, 13, 25] and serves to confirm the validity of both parameters. Secondly, students progressing from their first to their second year at medical school perceived increasing stress and again, this has previously and internationally been reported [5]. Unfortunately, we here observed – even though in the second academic year only – that the increase in perceived stress had shifted the students’ academic performance along the Yerkes-Dodson curve towards a less productive range [26]. Thirdly, and this again was to be expected considering the above reported connection, the increase in perceived stress was paralleled by a decline in mindfulness [5, 12, 13, 27].

In contrast, the lack of any correlation between mindfulness and academic achievements – assessed twice at T1 and T3 - was unexpected. Even though our findings confirm a previous cross sectional study on 289 medical students across all academic years, results from middle school children and a broad variety of bachelor students were more promising [12, 22]. However, the latter study relied on self-reported grade point averages and did not report on any time delay between exam and assessment of mindfulness [22]. Also, we cannot exclude that any impact of mindfulness on scholarly success is dependent on age or subject specialization.

Most importantly, we here showed that six two hours-interventions on MBSR impacted positively on the students and not only helped to preserve mindfulness and to contain the increase in stress, but also fostered scholarly success. Indeed, preclinical and clinical medical students have previously been shown to benefit from an intervention on MBSR, and reductions in psychological distress and perceived stress were paralleled by increases in self compassion, empathy, spirituality and mindfulness. While some of these effects were only measured at completion of the intervention and therefore do not allow to deduce any permanent effect [14–17], others were shown to last for up to 20 months [18–20]. We here assessed mindfulness and perceived stress six months after completion of our intervention and therefore confirm a medium-term impact of our intervention on these two parameters. However, the situation was different for academic achievements. Here a positive impact resulting from the intervention was short lived and only measureable at T2, shortly after completion of the intervention. Already at T3, any positive impact had tapered off. A previous publication discussed that repeated practice of mindfulness in private might lead to more lasting effects [18]. However, increased academic demand during the first two years at medical school or negligence may prevent students from taking the time for practicing formal mindfulness. To our knowledge, this is the first study to present a controlled analysis of MBSR and its longitudinal impact on academic achievements. We consider our results promising and would like to speculate, that continuous refresher seminars and follow-ups into the clinical years at medical school might strengthen mechanisms to deal with stress and improve scholarly success. Our results were the more astonishing as the students participating in the intervention started out with a significantly reduced mindfulness in the first term (Fig. 4). Even though we have no way of finding out retrospectively, it would be interesting to know if the students who volunteered for our intervention were aware that their mindfulness presented room for improvement or whether we are looking at a chance finding.

There are limitations to our study and they concern the study design. Choosing a randomized controlled trial instead of a non-randomized one may have rendered intervention and control cohorts comparable for mindfulness. However, forcing students to participate in a mindfulness-based intervention if there is no appreciation for the topic may be counterproductive [28]. More limitations of our study include the small sample size of the intervention cohort and the duration of both, the intervention itself and the observation period. However, the short term positive effects of our intervention on scholarly success combined with the preservation of mindfulness and containment of perceived stress after six months are encouraging and suggestive of follow-up studies to investigate if longer-lasting effects can be achieved if the formal techniques of mindfulness such as meditation and bodyscan are routinely trained until applied automatically.

## Conclusion

Perceived stress and mindfulness correlated inversely at both time points. The intervention on mindfulness based stress reduction helped to contain stress and maintain mindfulness during the observation period and this effect lasted for at least six months beyond completion of the intervention. In contrast, beneficial effects on scholarly success were transient and only detectable at completion of the intervention.

## Supplementary Information

**Additional file 1: Additional** **Table 1.** Validity is confirmed for PSS and MAAS; **Additional Table 2.** Item descriptive statistics PSS at T1; **Additional Table 3.** Item descriptive statistics MAAS at T1; **Additional Table 4.** Item descriptive statistics PSS at T3; **Additional Table 5.** Item descriptive statistics MAAS at T3

## Data Availability

The datasets used and analysed during the current study are available from the corresponding author on reasonable request.

## Abbreviations

PSS: Perceived Stress Scale
MAAS: Mindfulness Attention and Awareness Scale
