# Supplementary material for: Mindfulness-based intervention helps preclinical medical students to contain stress, maintain mindfulness and improve academic success
Source: BMC Med Educ. 2021 Mar 5;21:145. doi: 10.1186/s12909-021-02578-y (PMC7934360; doi:10.1186/s12909-021-02578-y)
Supplement: Supplementary file 1 — Additional file 1: Additional Table 1. Validity is confirmed for PSS and MAAS; Additional Table 2. Item descriptive statistics PSS at T1; Additional Table 3. Item descriptive statistics MAAS at T1; Additional Table 4. Item descriptive statistics PSS at T3; Additional Table 5. Item descriptive statistics MAAS at T3 [file 12909_2021_2578_MOESM1_ESM.docx]

**Mindfulness-based intervention in preclinical medical students: a strategy for containment of stress, preservation of mindfulness and improvement of academic success**

**Luisa Charlotte Lampe and Brigitte Müller-Hilke, PH.D., MME***

Core Facility for Cell Sorting & Cell Analysis, Laboratory for Clinical Immunology, University Medical Center Rostock, Rostock, Germany

***Correspondence:**Brigitte Müller-Hilke
email: brigitte.mueller-hilke@med.uni-rostock.de

Phone: ++49 381 4945883

Core Facility for Cell Sorting & Cell Analysis, Laboratory for Clinical Immunology, University Medical Center Rostock, Rostock, Germany

**Additional Table 1.** Validity is confirmed for PSS and MAAS

|  | CFI | TLI | Chi² / p-Value | RMSEA |
| --- | --- | --- | --- | --- |
| T1 PSS | 0.969 | 0.959 | 55.425 / 0.012 | 0.068 |
| T3 PSS | 0.989 | 0.985 | 43.380 / 0.130 | 0.043 |
| T1 MAAS | 0.954 | 0.947 | 123.786 / 0.011 | 0.053 |
| T3 MAAS | 0.939 | 0.929 | 142.042 / <0.001 | 0.063 |

Comparative-fit-index (CFI) and Tucker-Lewis Index describe incremental fits (ideally close to 0.95 or higher).
Root mean square error of approximation (RMSEA) describes absolute measures of fit (ideally 0.08 or smaller).

**Additional Table 2. Item deskriptive statistics PSS at T1**

| Item | In the last month, how often… | M | SD | rIT |
| --- | --- | --- | --- | --- |
| 1 | …have you been upset because something that happened unexpectedly? | 1.96 | 0.76 | 0.42 |
| 2 | …have you felt that you were unable to control the important things in your life? | 2.18 | 0.85 | 0.64 |
| 3 | …have you felt nervous and “stressed”? | 3.11 | 0.75 | 0.63 |
| 4 | …have you confident about your ability to handle your personal problems? | 1.53 | 0.73 | 0.63 |
| 5 | …have you felt that things were going your way? | 1.74 | 0.78 | 0.62 |
| 6 | …have you found that you could not cope with all the things that you had to do? | 2.67 | 0.87 | 0.63 |
| 7 | …have you been able to control irritations in your life? | 1.42 | 0.74 | 0.47 |
| 8 | …have you felt, that you were on top of things? | 1.75 | 0.70 | 0.68 |
| 9 | …have you been angered because of things that were outside your control? | 2.29 | 0.91 | 0.21 |
| 10 | …have you felt difficulties piling up so high, that you could not overcome them? | 2.48 | 0.98 | 0.68 |
| TS |  | 21.13 | 8.07 |  |

TS Total Score, rIT corrected item correlation

**Additional Table 3. Item descriptive statistics MAAS at T1**

| Item |  | M | SD | rIT |
| --- | --- | --- | --- | --- |
| 1 | I could be experiencing some emotion and not be conscious of it until sometime later | 4.05 | 1.04 | 0.49 |
| 2 | I break or spill things because of carelessness, not paying attention, or thinking of something else. | 4.76 | 1.22 | 0.46 |
| 3 | I find it difficult to stay focused on what’s happening in the present. | 3.89 | 1.21 | 0.60 |
| 4 | I tend to walk quickly to get where I’m going without paying attention to what I experience along the way. | 2.86 | 1.09 | 0.30 |
| 5 | I tend not to notice feelings of physical tension or discomfort until they really grab my attention. | 3.71 | 1.35 | 0.69 |
| 6 | I forget a person’s name almost as soon as I’ve been told it for the first time. | 3.42 | 1.52 | 0.09 |
| 7 | It seems I am “running on automatic,” without much awareness of what I’m doing. | 3.86 | 1.18 | 0.73 |
| 8 | I rush through activities without being really attentive to them. | 3.82 | 1.25 | 0.73 |
| 9 | I get so focused on the goal I want to achieve that I lose touch with what I’m doing right now to get there. | 4.11 | 1.23 | 0.64 |
| 10 | I do jobs or tasks automatically, without being aware of what I'm doing. | 4.18 | 1.14 | 0.71 |
| 11 | I find myself listening to someone with one ear, doing something else at the same time. | 3.47 | 1.33 | 0.61 |
| 12 | I drive places on ‘automatic pilot’ and then wonder why I went there | 4.68 | 1.39 | 0.63 |
| 13 | I find myself preoccupied with the future or the past. | 3.32 | 1.38 | 0.40 |
| 14 | I find myself doing things without paying attention. | 3.82 | 1.27 | .075 |
| 15 | I snack without being aware that I’m eating. | 4.45 | 1.59 | 0.49 |
| TS |  | 58.42 | 19.18 |  |

TS Total Score, rIT corrected item correlation

**Additional Table 4. Item descriptive statistics PSS at T3**

| Item | In the last month, how often… | M | SD | rIT |
| --- | --- | --- | --- | --- |
| 1 | …have you been upset because something that happened unexpectedly? | 1.95 | 0.88 | 0.35 |
| 2 | …have you felt that you were unable to control the important things in your life? | 2.34 | 1.07 | 0.80 |
| 3 | …have you felt nervous and “stressed”? | 3.24 | 0.97 | 0.70 |
| 4 | …have you confident about your ability to handle your personal problems? | 1.64 | 0.84 | 0.71 |
| 5 | …have you felt that things were going your way? | 1.81 | 0.92 | 0.71 |
| 6 | …have you found that you could not cope with all the things that you had to do? | 2.70 | 1.02 | 0.78 |
| 7 | …have you been able to control irritations in your life? | 1.35 | 0.75 | 0.71 |
| 8 | …have you felt, that you were on top of things? | 1.86 | 0.87 | 0.72 |
| 9 | …have you been angered because of things that were outside your control? | 2.64 | 0.99 | 0.61 |
| 10 | …have you felt difficulties piling up so high, that you could not overcome them? | 2.43 | 1.13 | 0.76 |
| TS |  | 21.95 | 9.45 |  |

TS Total Score, rIT corrected item correlation

**Additional Table 5. Item descriptive statistics MAAS at T3**

| Item |  | M | SD | rIT |
| --- | --- | --- | --- | --- |
| 1 | I could be experiencing some emotion and not be conscious of it until sometime later | 3.99 | 1.13 | 0.45 |
| 2 | I break or spill things because of carelessness, not paying attention, or thinking of something else. | 4.48 | 1.36 | 0.39 |
| 3 | I find it difficult to stay focused on what’s happening in the present. | 3.69 | 1.20 | 0.50 |
| 4 | I tend to walk quickly to get where I’m going without paying attention to what I experience along the way. | 2.53 | 1.22 | 0.50 |
| 5 | I tend not to notice feelings of physical tension or discomfort until they really grab my attention. | 3.47 | 1.39 | 0.59 |
| 6 | I forget a person’s name almost as soon as I’ve been told it for the first time. | 3.33 | 1.49 | 0.27 |
| 7 | It seems I am “running on automatic,” without much awareness of what I’m doing. | 3.35 | 1.23 | 0.73 |
| 8 | I rush through activities without being really attentive to them. | 3.18 | 1.28 | 0.74 |
| 9 | I get so focused on the goal I want to achieve that I lose touch with what I’m doing right now to get there. | 3.65 | 1.29 | 0.75 |
| 10 | I do jobs or tasks automatically, without being aware of what I'm doing. | 3.79 | 1.32 | 0.75 |
| 11 | I find myself listening to someone with one ear, doing something else at the same time. | 3.33 | 1.26 | 0.61 |
| 12 | I drive places on ‘automatic pilot’ and then wonder why I went there | 4.35 | 1.50 | 0.59 |
| 13 | I find myself preoccupied with the future or the past. | 3.17 | 1.25 | 0.43 |
| 14 | I find myself doing things without paying attention. | 3.57 | 1.23 | 0.75 |
| 15 | I snack without being aware that I’m eating. | 3.99 | 1.67 | 0.49 |
| TS |  | 53.89 | 19.82 |  |

TS Total Score, rIT corrected item correlation
